# CardioStart Online: A Virtual High School Tissue Engineering Course

**DOI:** 10.1007/s43683-023-00106-6

**Published:** 2023-03-20

**Authors:** Jasmine Naik, Anna Grosberg, Christine King

**Affiliations:** 1 Department of Chemical and Biomolecular Engineering, University of California–Irvine, Irvine, 92697 USA; 2 Department of Biomedical Engineering, University of California–Irvine, 3410 Engineering Hall, Irvine, 92697 USA; 3 Edwards Lifesciences Foundation Cardiovascular Innovation & Research Center and Center for Complex Biological Systems and The NSF-Simons Center for Multiscale Cell Fate Research, University of California, Irvine, Irvine, CA 92697 USA

**Keywords:** Tissue engineering, High school STEM, Online learning

## Abstract

In this paper, we altered an in-person high school tissue engineering program to create a virtual course. Through this alteration, we aimed to show that online programs can still be engaging and at the same time provide greater accessibility and flexibility to students. This was achieved through utilizing Google classroom as a virtual platform for students to engage with course modules and assessments. After analyzing pre- and post-program survey responses in both the in-person and online offerings of the CardioStart program, it was found that students improved in their understanding of all of the tissue engineering topics that were introduced in the programs. Furthermore, when comparing the results from the in-person versus online offerings of the program, it was found that the level of student understanding and learning of these topics was similar across the in-person and online programs. We were also able to engage five times the number of students online as compared to the in-person program, which was conducted yearly for six summers. However, many students indicated that their experience would have been better if hands-on activities were included to supplement their knowledge of cell culture techniques after completing the course. The online program improved accessibility and scalability of the program compared to in-person workshops. Future work will consist of bridging this virtual course and the hands-on experiments performed during the in-person program to provide interested students access to laboratory experiences.

## Motivation

As biotechnology and pharmaceutical companies push to advance current treatments for various ailments and diseases, such as a vaccine for COVID-19, Science, Technology, Engineering, and Math (STEM) related jobs, such as biomedical engineering, have been projected to exhibit more stable growth than other jobs.^1–4^ To fill these roles, the country will require a supply of STEM trained graduates to enter the workforce; therefore, youth STEM education is now more critical than ever.^5–7^ In the current job market, even as these careers surge, many people remain unemployed because they do not have a degree in STEM.^2^ The relatively small amount of STEM degrees can be attributed to a lack of encouragement, role models, and access to quality education.^3,8–10^ Education curricula content across the US varies significantly depending on the resources available, leaving some students lacking STEM exposure.^6,9,11,12^ Further, to bridge the gaps between fundamental knowledge and the ability to apply knowledge to real-world problems, students need access to engaging extracurricular programs and role models to emulate.^10,13^

To fill this need, many schools and communities provide after school programs. In a survey conducted by Afterschool Alliance, 200 households in each state were asked about afterschool program activity and the data collected was projected to the youth of America. The data collected from the survey revealed that around 50% of afterschool programs offer two or more days of STEM instruction in the hopes of inspiring the next generation of STEM graduates.^14^ In 2020, a new survey, conducted by the same group, found the number of after school programs with STEM instruction grew and they predict this trend to continue.^15^ Indeed, these programs have improved students’ attitude towards STEM fields and careers, increased STEM knowledge and skills, and increased the likelihood of student graduation and pursuing a STEM career; however, there is still an unmet need for afterschool programs as only 8.4 million students are enrolled, leaving more than 40 million students without programs to attend, and the number of students enrolled continues to decline as more barriers are faced.^15^ In fact, as many as 19.4 million students would sign up if a program were available.^14–20^ The main reasons for students not enrolling are program costs and a lack of program availability, both of which were cited to be higher in rural areas.^14,15,21,22^ Along with funding challenges, some programs are also not aligned with students interests, with only half of them offering stimulating STEM activities.^14,21^ In order to allow for both more rigorous STEM involvement and to reach even more students, universities are looked to for additional programs. As a result, universities typically offer summer programs to high school students that allow them to explore STEM topics as laboratories and classrooms are utilized during the school year by undergraduate and graduate students.

Many universities offer summer programs to increase STEM interest to high school students as they have the available research and laboratory spaces for students to explore their interests with more in-depth STEM integration than after school programs.^21^ Despite a seeming abundance of resources, the summer programs offered by many universities are limited to local students or are expensive due to a variety of program costs such as housing, personnel, and supplies needed.^19^ The costs associated with running these programs also limit the number of students that can attend each program, which increases competition to attend and lowers accessibility.^19,23,24^ Further, many engineering summer programs are not able to devote significant time to each engineering discipline, therefore omitting many topics that students may find engaging.^11,25–28^ To expose more students to biomedical engineering and more specifically tissue engineering, a summer program that is easily accessible and scalable is essential for inspiring the next generation of scientists and engineers.

In this paper, we describe a scalable online program centered on tissue engineering that was altered from our in-person program, CardioStart.^20^ Through the creation of a modular online platform, students from across the country were able to engage with Power points and virtual projects, even while the COVID-19 pandemic limited extracurricular activities. In the virtual format, we aimed to assess whether online tissue engineering programs and in-person programs promote similar learning opportunities, while being mindful of the fact that online programs have greater accessibility to students.

## Framework

**FIGURE 1:**
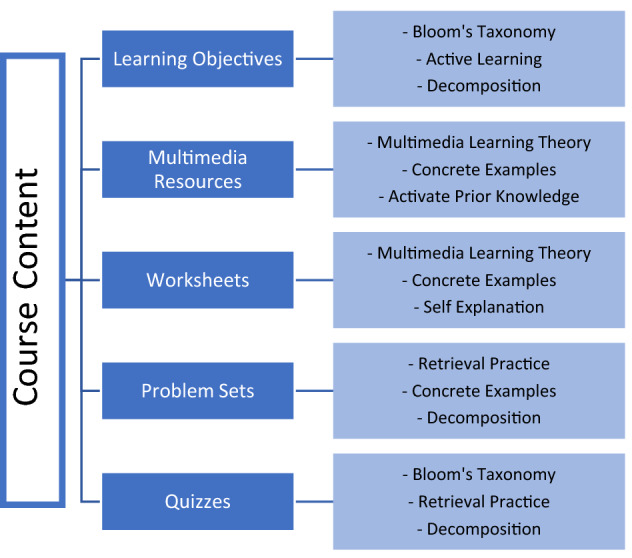
Evidence based educational methods used to design the course content of the CardioStart Program.

After researching multiple online platforms and classroom models, we chose to model the course using a MOOC framework, as presented in Fig. 1.^29^ Here, the dark blue boxes represent course content based on pedagogical strategies indicated by the lighter blue boxes. By adapting a MOOC framework to the CardioStart online program, we were able to combine best learning practices in the classroom and transfer them into an easy to use, online platform. Here, we detail each evidence-based practice method and how it was incorporated into the CardioStart online program.

### Utilizing Bloom’s Taxonomy to Develop Assessments of Learning Outcomes

Given the desired learning outcomes presented in Table 1, several higher levels of Bloom’s Taxonomy^30^ were targeted throughout the course. Learning outcomes included understanding ideas and concepts surrounding tissue engineering such as cell biology, scaffolding materials for cellular growth, and cell interactions with the environment. The concepts were introduced and then students applied this information to learn tissue engineering skills such as aseptic techniques to culture cells, evaluating cardiac beating to determine normal cardiac rhythms, and creating an experiment by developing a hypothesis given the learned knowledge of immunostaining, image analysis, and cell culture protocols.

**Table 1:** Student learning outcomes and targeted Bloom’s taxonomy levels^30^ of the in-person program vs online program.

Topic	In-person		Online	
	Student learning outcome	Bloom’s taxonomy level	Student learning outcome	Bloom’s taxonomy level
Aseptic technique	Students will be able to apply aseptic techniques to culture cells	Apply	Students will be able to recognize aseptic techniques to properly perform cell culture	Remember
Lab safety	Students will be able to use lab safety skills to mitigate harm while working in the lab	Apply	Students will be able to identify unsafe practices in the lab to mitigate harm while working in the lab	Remember
Passage cells	Students will be able to execute protocols to passage cells	Apply	Students will be able to describe protocols used to passage cells correctly	Remember
Count cells	Students will be able to execute protocols to count cells	Apply	Students will be able to describe protocols used to count cells correctly	Remember
Experimental design	Students will be able to design an experiment to test a hypothesis	Create	Students will be able to identify proper controls to design an experiment	Remember
Immunostaining	Students will be able to identify correct immunostains to detect multiple cell proteins	Remember	Students will be able to identify correct immunostains to detect multiple cell proteins	Remember
Cardiac stress	Students will be able to classify cardiac beating to determine normal cardiac rhythm	Understand	Students will be able to describe cardiac beating to determine normal cardiac rhythm	Remember
Live vs. fixed staining	Students will be able to recognize the difference between staining types to improve data collection	Remember	Students will be able to recognize the difference in staining types to improve data collection	Remember
Quantitative image analysis	Students will be able to design gif using ImageJ to understand scientific image analysis	Create	Students will be able to design gif using ImageJ to understand scientific image analysis	Create
Ethics	Students will be able to discuss biomedical research cases	Understand	Students will be able to discuss biomedical research cases	Understand
Scientific writing	Students will be able to compose an abstract for a scientific journal article	Create	Students will be able to compose an abstract for a scientific journal article	Create

### Student-Centered Learning Strategies Utilized in the Online Course

To teach each learning outcome presented in Table 1, the learning strategies developed for the online program were based on constructivist theory; the development of understanding requires the learner to actively engage in meaning-making.^31^ To this end, a student-centered learning approach was utilized.^32^ in which the learning modules were constructed such that the student was able to participate at every stage of the program while supporting their individual learning processes through student-centered work such as constructing knowledge, learning to learn, and making sense in the mind. This includes both active^33–36^ and inquiry-oriented learning,^37–39^ which emphasize interactions with peers and instructors through continuous activities and feedback so that they can apply their learning. These types of learning activities support the 5 phases of inquiry in the learning cycle: Engagement, Exploration, Explanation, Elaboration, and Evaluation.^40^

### Multimedia Learning Theory for Online Implementation

**FIGURE 2:**
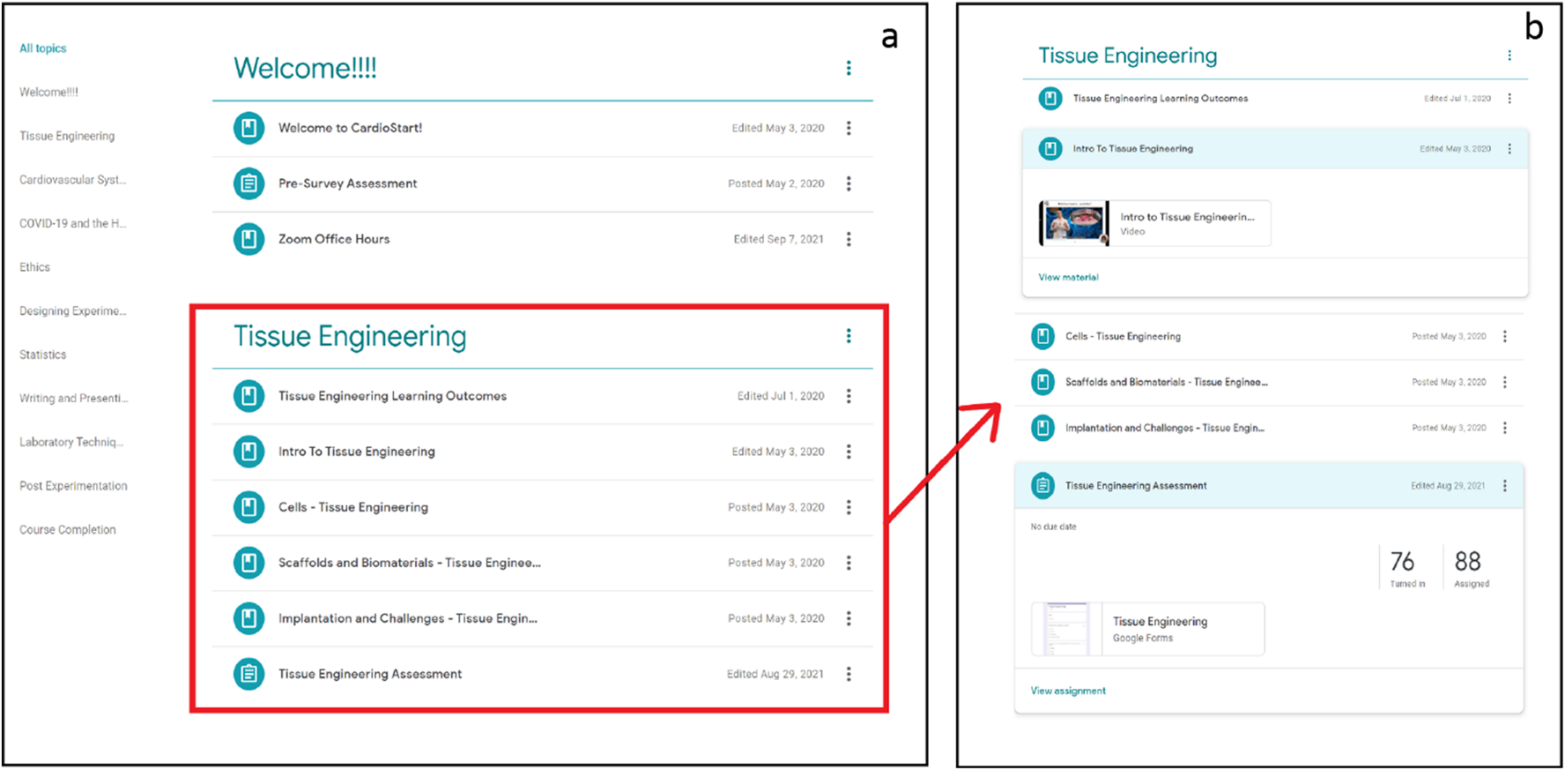
Google classroom setup of the online program. (a) Full classroom and topics. (b) Tissue engineering module example. Note that all modules included individual learning outcomes, power points, and a quiz.

Multimedia learning theory focuses on providing students multiple ways of understanding the same knowledge.^41^ The online program utilized PowerPoint presentations with voiceovers recorded to each slide. Handouts were also uploaded into the appropriate modules which provided examples. The online modules also had links to YouTube videos in each module to supplement the pre-recorded presentations. Due to the online structure, every module utilized multimedia learning theory heavily as all content was provided in different forms of media. An example of one online module can be seen in Fig. 2.

### Concrete Examples to Solidify New Knowledge

Concrete examples allow students to apply what they learned to a new topic or idea.^42^ In CardioStart, students were given many examples and asked to create something new from previous lectures and examples. Each module used concrete examples to solidify new knowledge by incorporating a principle students already learned. One of these projects was to write an abstract based on a research article they selected with the abstract removed. Prior to this exercise, students watched a presentation of abstracts and were given guidelines on how abstracts are normally written. They were then given examples of abstracts and the journal articles to which they belonged. They were then asked to use the guidelines given to write an abstract for a new journal article and submit in the corresponding module.

### Activation of Prior Knowledge to Connect Foundational Knowledge to New Knowledge

Activating prior knowledge allows students to use the foundations of what they know to understand more complex topics.^43^ Students who have enrolled must have taken biology, chemistry, and algebra before beginning the course. Some students had already taken AP versions of these courses as well. To accommodate multiple skill levels, we leveraged activating prior knowledge by including PowerPoint slides to the beginning of each new concept with more general concepts. As the modules progressed, the first few slides captured the important topics from previous modules to make it easier for students to build on previous information. Quizzes at the end of each learning module allowed students to both determine whether they had met each learning outcome presented as well as understand how previous material connected to the new module.

### Self-explanation to Improve Information Retention

Self-explanation was utilized throughout the course as each PowerPoint recording in the online course included stopping points that asked students to pause and think about questions that were asked before resuming the video. This practice has been shown to improve students retention of new information and is more beneficial than explaining after teaching new concepts.^44^ Each module had these points within them for students to think about everything they had learned to address the question within a new topic. For example, in the cardiac tissue engineering module, students were asked about challenges within the field. Students had to rely on the challenges presented in tissue engineering and apply that to the new topic, thus incorporating prior knowledge and inquiry-based learning.

### Retrieval Practice

The quizzes at the end of each learning module required students to utilize retrieval practice of the content provided for the desired learning outcome. Retrieval practice allows students to recall information from the learning module without having it in front of them through quiz questions.^45^ These learning module quizzes were low stakes as students could retake them as many times as they liked after checking what questions they answered incorrectly to practice their information recall abilities. After a quiz was submitted, students received feedback identifying which questions were incorrectly answered. The students then had the option of repeating the assignment to replace their score. For overall continuity, these questions were tailored to the overall learning outcomes of each module.

### Decomposition of Concepts to Understand Complex Learning Outcomes

As the overall course objective was to teach students about tissue engineering, decomposition was used to break this topic into smaller topics. For example, for students to understand how cell structure change over time and be able to track these changes for various tissue engineering applications, students were asked to create a gif using ImageJ and fluorescent images of cells that were provided. In order to complete the task, students were first taught in previous modules basic cell biology concepts such as cellular structure or architecture. In other previous modules before this assignment, they were also taught how to select immunostains to be able to observe multiple cell structure simultaneously during imaging of cells using microscopy. By recalling information from these previous modules and applying new knowledge of ImageJ software analyses such as boundary analysis, students were able to create gifs of cells in multiple colors to see the various structural changes. By decomposing this project into smaller, easier to understand parts, students were able to develop and create a final project capable of being directly applied to tissue engineering applications.

## Methods

To achieve a MOOC framework during the online program offering, we chose to use Google Classroom^46^ as it is widely accessible for all students and free of charge. Through this platform, modules were adapted from the previous version of CardioStart,^20^ thus keeping the content the same while adding training videos where in-person laboratory procedures would normally be performed. The Google Classroom consisted of modules covering the following topics: introduction to tissue engineering, cardiovascular system, ethics, experimental design, statistics, writing and presenting scientific work, laboratory techniques, and image processing (Fig. 2a). Within each module (Fig. 2b), students could view recorded power point presentations, collections of engaging YouTube videos to provide real world references, journal articles which aided in the completion of small projects, and discussion boards (see Framework for additional information). The course was run asynchronously to allow flexibility and greater accessibility. To further engage with students, graduate students offered synchronous office hours twice a week on Zoom at various times in the afternoon for enrolled students to ask questions they may have about the material covered.

To implement the student-centered learning strategies mentioned in the Framework section, the following inquiry-based learning strategies were utilized in the program. These strategies focused on requiring students to follow methods and practices utilized by scientists to discover new causal relations by allowing students to formulate hypotheses and test them by conducting experiments and make observations virtually. Activities that supported this type of learning strategy included performing case studies to engage in the lecture material and draw conclusions given different desired hypotheses (e.g. ascertain which stem cell type to use given a scenario and ethical considerations), self-explanation and reflection during pre-recorded lectures given their observations through the lecture, and performing an experimental-based approach to exploring and evaluating cell structure through image analysis given imaged cells.

In addition to the above inquiry-based learning approaches, active learning strategies were also incorporated throughout the program. Techniques such as case studies that incorporated virtual discussions required students to interact together on discussion boards to comment on one another’s responses to complete virtual assignments. For instance, during an ethical considerations case study on understanding different stem cell types and different scenarios for when each type is used in tissue engineering applications, students were required to work through virtual discussion boards together by commenting on each other’s responses to interpret a given case and decide which stem cell type would have the most ethical resolution. This learning strategy uses a problem-based learning strategy similar to “think-pair-share” in that it required students to think to themselves on a topic and then pair up to discuss it via a virtual discussion board prior to submitting their response.^47^ After the case studies and the pre-recorded lectures were performed, students also engaged in the material through problem-based quizzes in each learning module that provided immediate feedback to the learner, a strategy similar to personal response systems (e.g. clicker questions) that are typically utilized in in-person lectures to promote active learning in the classroom.^48^ All these strategies were implemented in each consecutive learning module throughout the online program.

To gauge student knowledge before, during, and after the program, multiple quizzes were built into the course. These learning module quizzes were completed in both the in-person and online offerings of the course. Before beginning the course and after course completion, students also completed a pre- and post-survey to assess their overall knowledge of tissue engineering concepts as well as assess the program’s effectiveness to teach these topics after the completion of the course. Throughout the course, each module also contained a short quiz, which could be taken multiple times until the students were satisfied with their score. These assessments allowed students to evaluate their knowledge at the beginning of the program, what they learned after the completion of each learning module, and what they mastered by completing the CardioStart program. The analysis and publication of the cumulative results of these assessments were approved by the Institutional Review Board at the University of California Irvine IRB No: 2020-5964. To this end, an independent samples *t*-test was performed on the assessments that were provided after each learning module to compare the achievement of learning outcomes in the online and in-person offerings of the program. An independent samples *t*-test was chosen because different cohorts of students performed the online and in-person CardioStart programs, and a *z*-test confirmed that the ordinal assessment data came from a normal distribution.

Local schools were emailed to inform students about the online program. This included all local schools regardless of their population and demographics, as all schools near the institution were included and none were targeted based on their demographics. Because of this, the program consisted primarily of local students not belonging to minority or Title 1 schools. Students from these targeted schools were recruited by sending IRB approved flyers to the head of the science department at each of the schools in the neighboring districts. Interested students then emailed the CardioStart program director, who then asked students to submit IRB consent and assent forms to participate in the CardioStart research study. Students who did not submit these forms still participated, however these students’ pre- and post-program surveys to assess the program’s effectiveness were not utilized in the analysis of the program’s effectiveness and all of their data were removed from the study.

## Results

### Student Recruitment

**FIGURE 3:**
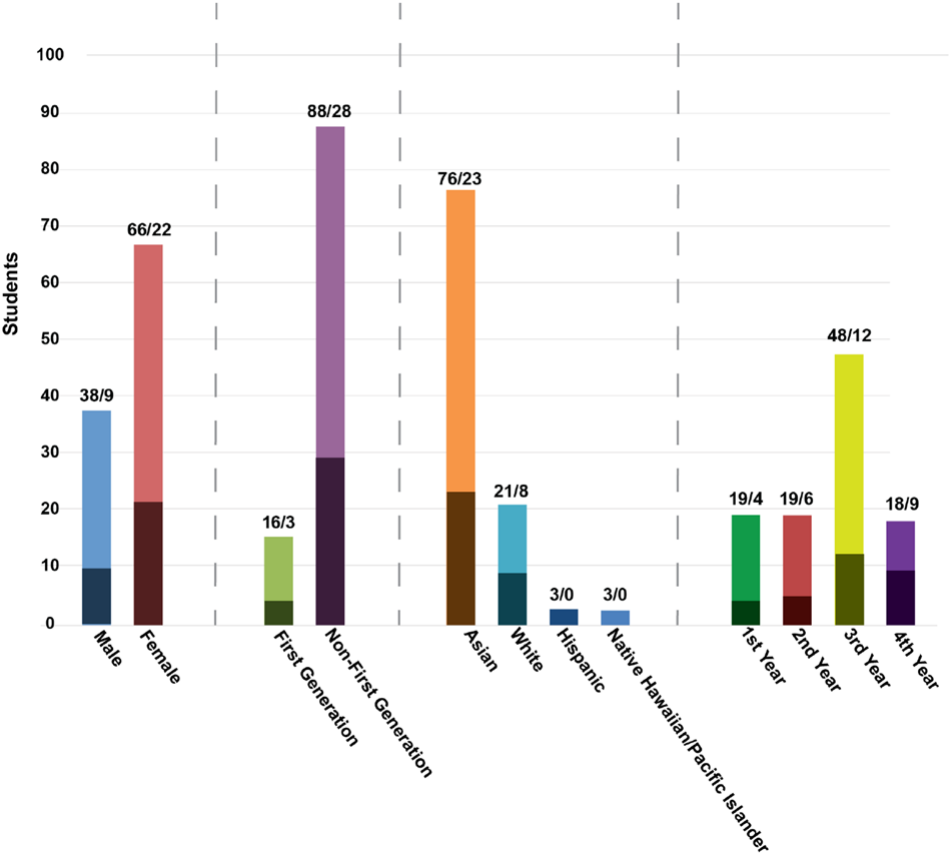
Demographics of enrolled students (light colored bars) and students that completed program (dark colored bars): male vs female students enrolled, first-generation vs non-first-generation students, ethnicity of students, year in high school. Darker colored bar indicates students that completed program, numbers of total/completed are located on the top of each bar. Data is for online students only. *Data collected from July 13th, 2020, through October 20th 2021.

**FIGURE 4:**
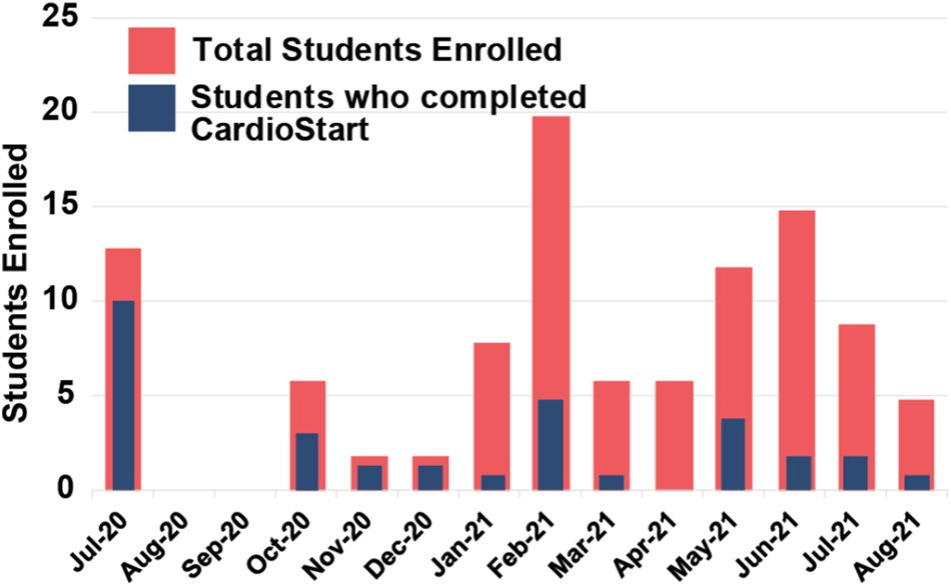
Total students enrolled per month and student’s completion of program. *Data collected from July 13th 2020 through October 20th 2021.

During this study, 118 students enrolled in the online CardioStart program and 104 consented to have their data used for research purposes. Of these students who consented to participate, 31 completed the course when this article was prepared. In comparison, 18 students participated in previous in-person CardioStart programs (Table 2). Due to IRB constrictions of the in-person cohorts, no data was collected on student demographics. While 31 students completed the course, the remaining students participated as much as they desired (Fig. 3). Students were also able to join the program throughout the year instead of being limited to summer months (Fig. 4). Due to IRB restrictions, demographic data was not able to be tracked based on when students enrolled.

**Table 2:** CardioStart iterations, the number of students that participated, and years data collected.

Program	Number of students enrolled	Number of students completed	Years run
6-Week CardioStart	14	14	2014–2016
3-Week CardioStart	4	4	2019
Online CardioStart	104	31	2020–2021

Note that in person data was presented in a prior publication of the CardioStart program^20^

### Cost of Running the Programs

**FIGURE 5:**
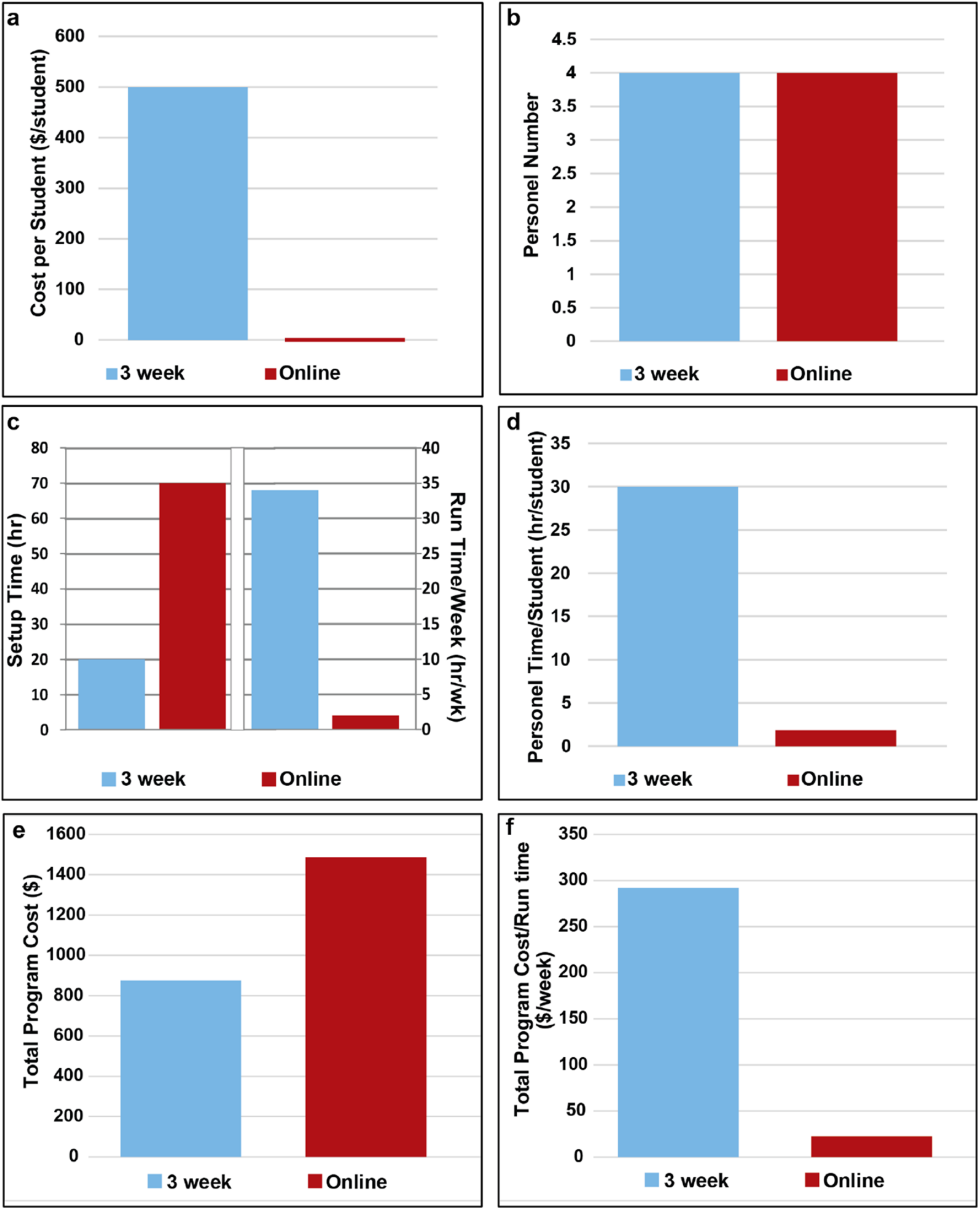
Program costs comparing the online and in-person offerings of the program. (a) Cost per student in 3-week program vs online program. (b) Personnel number required for the 3-week program and online program. (c) Personnel Time required for setup and hours/week. (d) Personnel time per student for the 3-week program and online program excitement total overall program cost. (f) Total overall program cost divided the total duration the program was offered. *Data collected from July 13th 2020 through October 20th 2021.

One of the main barriers to having students participate in these programs are the associated costs to the universities and students. To evaluate this barrier, we analyzed the expenses of the online CardioStart program and compared it to the three-week in-person program (Fig. 5).^20^ The online program supply cost per student was $0 as Google Classroom was free to access, and no experimental supplies were required for students, unlike the 3-week programs, which cost $500 per student (Fig. 5a). Indirectly related to cost when considering personnel required salary to run the program, the total number of required personnel (Fig. 5b) was equivalent as both the online program and the three-week in-person program required two instructors and the support of two academic advisors. However, the overall time spent by personnel in program setup is greater in the online program (Fig. 5c). The initial startup time for the online program was 70 h for content creation while the 3-week in-person start up time was 20 h. In contrast, the online program had a greatly reduced run-time per week as 2 h of course maintenance was performed per week, while the 3-week in-person program required personnel to meet with students for 25 h each week. Moreover, the online program significantly reduced the total personnel time spent per student as there was no cap on student enrollment and thus made the online program more efficient (Fig. 5d). Finally, when considering the overall total program cost associated with each program offering type (Fig. 5e), the online program was more expensive than the in-person program, however, when divided by the total duration that the program was offered (i.e. “run time”), the online program was significantly cheaper to run than the in-person program (Fig. 5f).

### Pre- and Post-program Survey Comparison

**FIGURE 6:**
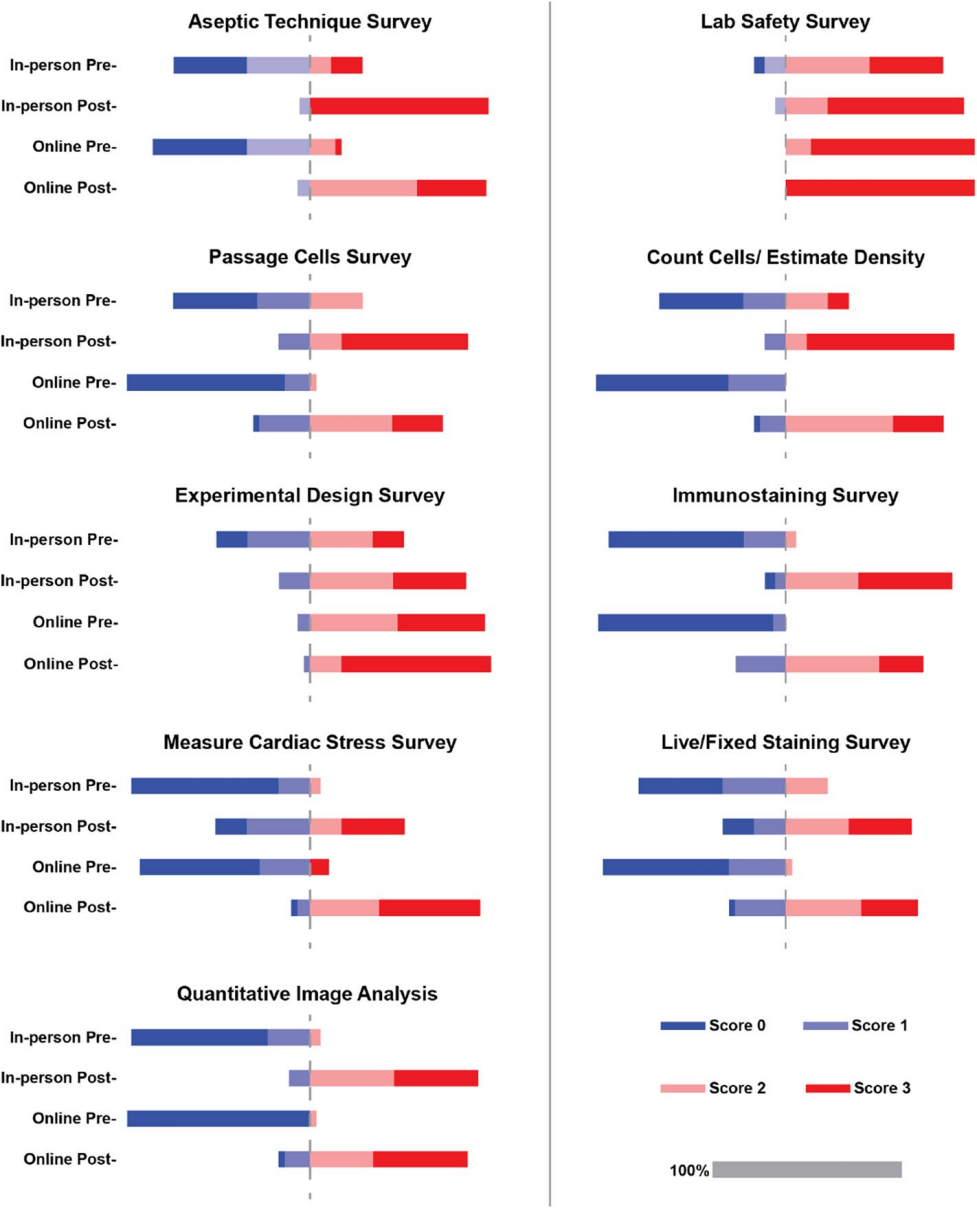
Pre- and post- survey response comparisons in the 3-week and online programs. Scores: 0—students never heard of the topic, 1—students heard of the topic, 2—students were familiar with aspects of the topic, to 3—students understand the topic well. The full bar is 100% of all the scores, and the length for reference is shown in the legend in gray. The gray dashed line represents the change from “unfamiliar” to “familiar or understanding” of the learning outcome. *Data collected from July 13th, 2020, through October 20th 2021.

For those students who consented, we were able to analyze their participation in the program. To determine how effective the online version of CardioStart was, the pre- and post-survey responses from the online program and the three-week in-person version were compared (Fig. 6). Students were self-evaluated through Likert-based survey questions on a scale from 0—students never heard of the topic, 1—students heard of the topic, 2—students were familiar with aspects of the topic, to 3—students understand the topic well. Questions were posed at the understand level on Bloom’s taxonomy on how well they could identify and discuss each topic for easy comparisons. However, the in-person cohorts learning objectives exceeded understanding unlike the online cohort. Table 1 compares Bloom’s Taxonomy levels for each topic covered. Score results for understanding the topics in both the online and in-person cohorts illustrate a shift from an unfamiliarity blue (score of 0–1) in the pre-survey to an understanding red (score of 2–3) in the post-survey in every topic after completing CardioStart (Fig. 6). Overall, the survey results indicate that this trend exists regardless of whether they participated in the online or three-week in-person version of CardioStart.

### Assessment Scores

**FIGURE 7:**
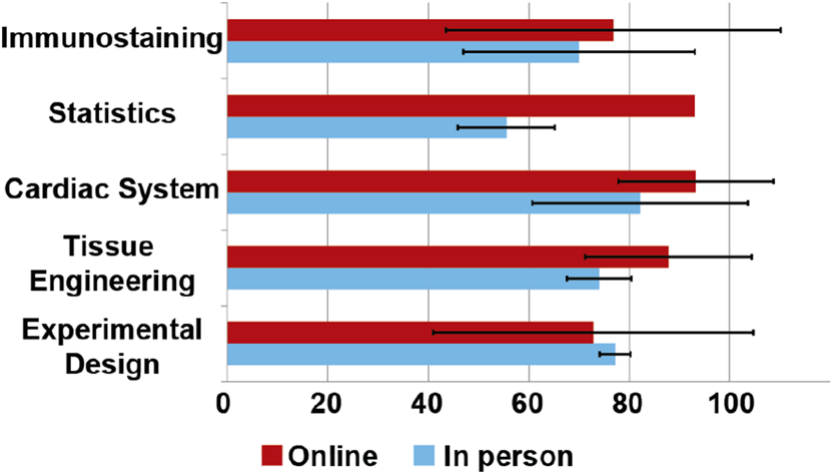
Assessment scores of the 3-week vs online program. *Data collected from July 13th, 2020, through October 20th 2021. Number of students included in each average for the online course was 41, 43, 59, 65 and 43, respectively, while the number of students for the in-person course in each category was 4. Error bars reflect standard deviations, *t*-test performed, and significance found if *p* < 0.05.

To determine how well each module translated to the online platform, we performed a comparison between the average assessments scores from the online version of CardioStart and the three-week in-person version. These assessments were short quizzes given after the completion of each learning module and scored out of 100. Students were able to retake the quizzes indefinitely with the highest score being reported. The averages below indicate each students’ best score. When comparing the two programs, there is no significant difference in the outcomes between the three-week in person and online programs (Fig. 7).

### Student Assessment of the Program

To more comprehensively evaluate the program, quantitative questions were asked on topics CardioStart covered, and responses were collected. Students were asked to give survey questions a score of 1—CardioStart did not address this topic or strongly disagree with the statement to 5—CardioStart greatly addressed this topic or student strongly agrees with the statement. Student responses were overall positive and can be seen in Table 3.

**Table 3:** Quantitative student responses to skills learned through the CardioStart program.

	Mean	Median score	Standard deviation
Helped my understanding of scientific research	4.24	4	0.73
Develop knowledge about the cardiac system	4.45	5	0.72
Understand difficulties of entering research career	4.66	5	0.54
Strengthen skills as scientist	4.28	4	0.78

Scores: 5—CardioStart greatly addressed this topic, 4—CardioStart addressed this topic, 3—CardioStart mentioned this topic, 2—CardioStart spent little time on this topic, 1—CardioStart did not address this topic

^*^Data collected from July 13th 2020 through October 20th 2021

Along with these questions, students in the online course were also asked open ended qualitative questions where they expanded on what they enjoyed most about the program and what could be improved:

I found learning about tissue engineering and the cardiac system something that interests me, and I therefore went about researching these topics in more detail for fun.

The course was very informative and the assignments were helpful in reviewing the content.

This program has allowed me to expand my knowledge especially in tissue engineering, the cardiovascular system, and statistics.

All of the information, presentations, and assignments really helped me learn about various aspects of cardiac and tissue engineering.

In addition to the above comments, several students also mentioned in the post-program survey that they enjoyed shorter videos that were only around 5–10 min long. Few mentioned that it was helpful that each video focused on only one to two student learning outcomes and included captions. On the other hand, many students commented that they had to rewatch the longer videos (those > 10 min) many times as their attention drifted or too much information was covered without a break.

Students were also invited to provide feedback on areas they would like improvement on:

Although I felt that this program was great online, the only improvement I would suggest is to incorporate more interactive elements. Some aspects, like the Fiji project, were engaging, but it would be more fun if there were hands-on experiences and live exposure.

I may have been better prepared for CardioStart if I had some background in advanced cell biology. A background video or vocabulary list introducing commonly used terms relating to tissue engineering would have been helpful.

## Discussion

In this work, we described the implementation of a scalable, online program for high school students, online CardioStart, which when compared to a similar in-person program, created greater accessibility (Fig. 2), scalability (Fig. 3), flexibility (Fig. 4), and increased overall cost effectiveness over time (e.g. Fig. 5f) while maintaining comparable effectiveness of achieving the desired learning outcomes. As seen in Fig. 3, due to the flexibility of the online format, CardioStart online was able to enroll a variety of students across all years of high school. This format allows the program to be taken by schools across the nation, rather than limiting those who are able to physically attend the campus. In addition, when comparing the program offerings, students were able to achieve the learning outcomes for the respective levels of learning in the in person and online versions of the program (Table 1), as well as were able to maintain the same level of perceived interest in pursuing STEM disciplines in the online offering as the in-person offering.

Through the use of multimedia, handouts, online quizzes, and pre- and post-surveys on the program’s effectiveness to teach tissue engineering concepts, we were able to compare the online program to the previous held in-person programs (Figs. 6 and 7). In comparing the two courses, both groups of students entered with the same relative knowledge and left the program more knowledgeable in all topics, regardless of the type of program offering (i.e. in-person vs. online). Students also felt the program provided them with the skills needed to become a successful scientist in the future (Table 3).

Generally, programs like these introduce students to topics not covered in high school curricula; however, one deficiency is that they are not widely available to many students.^19,23,24^ This lack of availability has become more evident due to COVID-19 closures, as students’ education has been significantly impacted.^7^ Through the creation of an online program, many additional students are now able to participate in a tissue engineering course that would not have been available to them due to limited accessibility or high program costs.

Compared to previous years when only an in person CardioStart program was offered, the online program allowed many students to participate as there were no space constraints (Fig. 3 and Table 2). The online platform also allowed students from all over the country, especially for students who are in schools with low college readiness scores. In future offerings of the online program, these schools will be targeted to encourage participation from underrepresented groups. Another benefit was increased flexibility, as students could join throughout the year instead of just during summer (Fig. 4). This flexibility also extended to the course as the course ran asynchronously allowing students to learn whenever they were available.

By utilizing the Google classroom, we experienced a 12-fold increase in student participation as we were not limited by lab capacity. The Google classroom can hold 1000 students and those who have completed the course can be removed to make space for new participants. Additionally, Google classrooms can be duplicated to accommodate more students. To make the program even more scalable, more research groups can develop similar programs with different modules, allowing all student interests to be addressed. In addition to the increase in students able to participate, we also must consider the number of personnel needed to run the program with the additional students. With the program running asynchronously with the exception of synchronous office hours, the personnel requirement should remain constant. If more interaction is required, additional office hours may be needed but can be offset by additional discussion boards or the addition of live group chats.

CardioStart online is also more scalable than the in-person program because of the reduction of overall costs associated with running the program over the duration of time it is offered. In particular, as seen in Fig. 5e and f, while the online program’s overall cost required to set up the program is more than the in-person program, the cost associated with running the program over time is much lower, as the program can be offered asynchronously throughout the year without the need for significant personnel costs to run it. The online program cost is also stable over time even as the program grows, as additional office hours can be performed asynchronously. While reducing program cost is important, the overall goal is to reduce program cost for students to perform the program. Due to the reduced space and overhead costs to run the program online to more students, many universities can offer financial support during program setup without needing to continuously financially support the program over time. This savings can then be transferred to students by lowering student program costs or even allowing students to enroll free of cost. As a result, online CardioStart allows for greater enrollment to students who may not have been able to pay program costs, thus improving its scalability and ability to target underrepresented groups to become more interested in tissue engineering and STEM careers.

As previously mentioned, the online program also allows for greater student flexibility, which we believe leads to increased student enrollment and thus increases scalability. In previous in-person programs, many interested students could not enroll due to school or extracurricular activity conflicts. Through the creation of self-paced modules, students had the flexibility of completing modules when they had time. As seen in Table 2 and Fig. 4, many more students were able to participate in the online program and thus engage with the content than previous in-person programs. Due to the modular format of online CardioStart, students had the option of choosing the modules that interested them. In comparison to traditional MOOC courses where uninterested students drop out, many CardioStart modules do not build on one another so students can jump modules. This is highly beneficial as students had the freedom to learn what they enjoyed, instead of being locked in a program for an entire summer while they were only interested in parts of the program. Despite allowing students to perform only the modules they were interested in the online program offering, the 31 students who completed the program is an approximate fivefold increase to what our lab could host for the past six years in any given summer.

In addition to increased scalability, we believe the online program also made CardioStart more accessible. Here, we define accessibility as greater student enrollment with fewer barriers to entry. Because of the IRB limitations to the in-person cohort, we are not able to distinguish the difference in student demographics between the in-person and online groups. However, based on the differences of student enrollment, we believe the online recruitment effort may result in a more diverse student population than would be possible with the small cohorts admitted to an in-person program (Fig. 3). This is due to the fact that in-person programs are limited to the demographics of the local area while online programs are able to attract students from a broader area. To include a more diverse population in future offerings of the program at our institution, low-income high schools that identify as Title 1 schools across the nation will be targeted. This will allow students an opportunity to learn a curriculum they might not have access to and is possible given the online format of the program.

While the program became more accessible due to no cost constraints as noted by the increase in students able to participate, the use of multiple teaching strategies such as videos, handouts, material repetition and simple course design was also implemented to allow the program to be more inclusive of different student learning styles (Fig. 2). For instance, the addition of subtitles for videos and text to explain images where necessary also improved inclusivity to those with learning disabilities. To improve flexibility, students were also given multiple attempts for each learning module quiz and submission assignment, and no assignment had deadlines attached. Office hours were also offered for students to ask questions. While the office hours provided were synchronous and students were able to participate, many could have been unable to join due to time zone restrictions. To accommodate all students, questions could be emailed as well, but students lost the interactive nature of office hours. In comparison to in-person courses, many of these considerations are based on individual student’s needs.

While there are many benefits to converting programs to an online format such as increased scalability and accessibility as discussed, there is a danger of sacrificing student learning. For the CardioStart online conversion, based on pre- and post-program survey comparisons seen in Fig. 6, students were able to understand the same concepts regardless of the virtual format. However, higher levels of learning are lost in the online format as seen in Table 1. In particular, the following learning outcome topics were notably lower, in the remember level of Bloom’s taxonomy, in the online format compared to the in-person format where these outcomes focused on the apply, create, or understand levels of Bloom’s taxonomy: aseptic techniques, lab safety, passage cells, count cells, experimental design, cardiac stress. This is further validated when students commented they wanted more hands-on activities. When comparing with other programs that converted to an in-person program into an online format, many of the same benefits and shortcomings were corroborated. One program stated that many more students were able to participate in the program, however, student engagement was lacking as many students struggled with virtual group conversations.^50^ Another such adapted program stated the same benefits of reaching more students due to the non-existent cost barrier and the ability to provide the program to more students.^51^ Furthermore, another program cited similar benefits of increased participation as online platforms can reach students across the country and more accessibility due to no program costs.^52^ However, this program cited the biggest challenge was the technology as students needed Zoom to participate as well as specialized programs to be used simultaneously.^52^ While there are many benefits to online instruction, many feel that post-COVID, re-introducing hands on experiments would be a great complement to the online program, and students that expressed an interest could be invited to the university to complete their training.^52^ Another solution would be to provide students with kits to conduct at-home experiments or providing a virtual lab component to the online classroom.

In the future, we will continue to improve the online CardioStart program by adding modules as topics become relevant. In the fall of 2020, we included a module on COVID-19 and the heart as research became available. This demonstrates that the modular platform described in this paper allows for continuous improvements to be made and for content to remain relevant to students. During the next program offering, we aim to merge the in-person and online CardioStart programs, which will address students’ comments about their desire to work in a tissue engineering lab. Students will first complete the online CardioStart course and learn the basics of the tissue engineering field. Interested students can then join a lab for cell culture training, experimental design practice, and to learn more about day-to-day life as a researcher. With this pipeline in place, we can then partner with other universities to leverage lab spaces across the country in order for students to complete the hands-on training. With the creation of online CardioStart, we hope that more universities will adopt similar programs to close the 19 million student gap of those who do not have access to STEM programs.^14,20^

### Application to Practice

There are many advantages to online learning as it increases accessibility, is cost effective and offers more flexibility than in-person programs. However, traditional disadvantages of online learning include longer time to provide feedback to individual students (rather than as a group in a classroom setting) and more prep time. In our design of CardioStart, these disadvantages of online learning have been alleviated by grading the learning module quizzes instantaneously for immediate feedback. Furthermore, while set-up time is substantial, when looking at the time required to run the course throughout the entire program, the set-up time for the online program offering becomes insignificant when compared to the in person program offering, as shown in Fig 5c. Many students also commented on the improved flexibility as they could continue other extracurricular activities simultaneously and enjoyed the office hours for personal feedback and growth even though they were offered online. In future offerings of the program at other institutions, we suggest implementing virtual office hours throughout the program offering to allow for personalized teaching and student feedback. We also noticed that many students took advantage of the module format in the online program offering, as students completed the modules they were interested in as opposed to having to complete the full course. In addition to implementing these advantages of our program at other institutions, there are further improvements that should be made in the design of the course given the findings throughout the development and analysis of the program offerings. The following sections describe these suggested improvements and best practices that should be followed when offering the CardioStart program at other institutions.

### Delivery of Learning Outcomes

As mentioned in the Results section, students enjoyed shorter videos that were around 5–10 min long that focused on only one to two student learning outcomes and included captions. In future offerings of the program at other institutions, instructors should provide short videos as well as a summary slide that summarizes key points in the videos to reinforce the information that was disseminated in the videos. The video content should also be kept short and should have activities provided between each video for students to perform retrieval practice and better understand the complex learning outcomes that were presented. Lastly, we recommend instructors to have the first presentation present a broad overview of what will be covered and define any new vocabulary that will be used in the module. This will allow students a chance to think about what is to come and reference back to that during the module.

Post-program analysis also showed that students enjoyed the module format where videos were organized by topic with post-learning module quizzes directly after the related content. Future offerings of the program at other institutions should consider this format, and organize the modules such that the first PowerPoint is based on foundational knowledge and the subsequent videos introduce new topics. Furthermore, the first module should provide the information needed for subsequent modules, and each subsequent module should be independent to allow students to be able to choose topics that they are interested in as opposed to completing the full course.

### Course Activities

Students had mentioned during office hours of that online program that they would like to have more interactions between other students such as group assignments. Future offerings of the online program at other institutions should consider adding more group assignments that can be done via the online discussion board, as the student’s favorite modules were those that included mandatory discussion boards or submission of their writing. For example, case studies in the ethics module received many responses during the online program offerings, as students utilized discussions boards to share their ideas of controversial topics while commenting on others’ opinions. In addition, the academic writing module that asked students to choose a journal article (provided with abstracts removed), write, and submit their own abstract also received many responses from students. Future writing modules in the program should provide abstracts from journal articles that are based on topics seen in current events and previous modules, and are also short and easy to understand. Lastly, results from the post-program survey found that students enjoyed commenting on other student’s projects and using previous projects posted on discussion boards to inspire them. In future program offerings, this module should include a discussion prompt that requires peer review of other student projects. Lastly, to better capitalize on online learning, discussion boards or other form of student interaction should be added to each learning module in future offerings of the program. Active learning online activities such as brainstorming via discussion boards, asking students to submit mind maps, performing a muddiest point, or providing discussion boards for teams to work on a group will better improve student interest and retention of the desired learning outcomes of the program.

### Assessments

To provide students with immediate feedback, as this can be challenging in an online format, the quizzes that assessed the students’ learning of each module were provided immediately displayed which questions were answered correctly and incorrectly after taking the quiz. By allowing students to retake these quizzes as many times as they would like as low stakes assignments, students were able to assess and correct their knowledge of the learning outcomes for each module, providing a formative assessment^49^ of understanding for each learning module. Furthermore, the small projects were implemented to achieve higher levels of Bloom’s taxonomy^30^ than “remember” or “understand” within the learning outcomes, such as “apply” or “create”. As previously mentioned, these small projects, such as creating a gif using images of cells and ImageJ, can provide instructors in future program offerings with summative assessments^49^ of students’ ability to build upon the learning outcomes towards a real-world application of their knowledge in tissue engineering. Future offerings of the online course at this institution and other institutions should consider both formative and summative assessments,^49^ as these assessment types compliment the determination of a student’s level of success or proficiency of tissue engineering learning outcomes, as well as allow students the opportunity to think critically as they apply their understanding under novel conditions to solve new problems.

## Data Availability

Not applicable
